# The Perception of the National Traceability Platform among Small-Scale Tea Farmers in Typical Agricultural Areas in Central China

**DOI:** 10.3390/ijerph192316280

**Published:** 2022-12-05

**Authors:** Yatao Huang, Hua Liu, Xuanxuan Guo, Wenxian Jiao

**Affiliations:** 1 College of Economics and Management, South China Agricultural University, Guangzhou 510642, China; 2 College of Geography and Environmental Science, Henan University, Kaifeng 475001, China; 3 Key Laboratory of Geospatial Technology for the Middle and Lower Yellow River Regions, Henan University, Ministry of Education, Kaifeng 475001, China

**Keywords:** agricultural product safety, national traceability platform, small-scale tea farmers, perceived differences, Q methodology

## Abstract

As one of the key technologies to ensure the safety of agricultural products, the national traceability platform is being widely promoted in China. However, it has not yet been widely adopted among farmers, especially small-scale farmers. Farmers are both producers and direct participants in the traceability of agricultural products. Their perception directly affects the effectiveness of the promotion of the national traceability platform. This study explores the perception of the national traceability platform among small-scale tea farmers in typical agricultural areas in central China. This research employed Q methodology, an approach that integrates both qualitative and quantitative data allowing individuals’ subjective understandings of a specific topic to be studied. The Q-sort procedure was performed in the field with 16 small-scale tea farmers. Next, Q-factor analyses were conducted using the Ken-Q analysis. The results show that small-scale tea farmers have different perceptions of the national traceability platform. Their main characteristics are active participation, resistant participation, risk aversion, and being driven by pressure. These four categories covered 52% of the perceived variance. Meanwhile, there is also a degree of internal consistency in the perception of small-scale tea farmers. Specifically, they are all concerned that participating in the national traceability platform may increase the cost and risk of cultivation and that it is difficult to obtain support from agricultural technicians. Therefore, understanding the perceptions of tea farmers of the national traceability platform is the premise for formulating effective promotion policies. Our research sheds light on the decision-making mechanisms for small-scale tea farmers to participate in national traceability platforms, further expanding the scope of current research on farmer behavior. This research has reference significance for promoting national traceability platforms in China and other countries around the world.

## 1. Introduction

Tea is the most widely consumed beverage in the world, second only to water [1]. The demand for tea has risen sharply over the past two decades and will continue to grow in the next decade [2,3]. More than 69% of the global population drinks tea every day, with an estimated daily consumption of 18–20 billion cups [4]. As a non-alcoholic stimulant beverage, tea has unique bioactivity and health benefits [5]. In recent years, the tea cultivation area in the world has expanded significantly due to the increase in the number of small-scale tea farmers [6]. About 70% of the world’s tea is produced by small-scale tea farmers, who constitute an important part of the global tea industry [7]. However, the low profitability of small-scale tea farming has become a serious threat to the sustainable development of the tea sector [8]. To achieve higher incomes, many tea farmers rely heavily on pesticide and fertilizer inputs to achieve higher yields [9,10]. This will not only damage the environment in the long run but also pose a great threat to human health [11,12]. Therefore, with the increase in tea consumption, the importance of tea safety has become increasingly prominent [13].

In order to ensure the safety of agricultural products, traceability systems have been established in many countries and regions around the world. However, such systems are mostly distributed in developed countries, such as those in the European Union [14], the United States [15], South Korea [16], and Australia [17]. The application of traceability systems in agriculture has not yet been fully established, especially in developing countries. India has established a traceability system, not for the purpose of tracking but rather to display the prices of goods [18]. The traceability system in Malawi is ineffective because the food supply chain is not traceable [19]. The traceability system in the Taiwan region of China also failed to achieve the expected success due to the low participation rate of farmers [20]. Meanwhile, traceability systems are still in the early development and application stage due to the influence of blockchain and the Internet of Things (IoT) [21]. In addition, due to restrictions from government regulations, unstable food safety policies, unorganized market areas, inadequate supply chain facilities, lack of cold chain facilities in the field, and insufficient agricultural practices by a large number of farmers, the usage rate of traceability systems is still relatively low [22].

Affected by individual food safety incidents in China, consumers are constantly seeking healthier and safer food [23]. Several Chinese government departments have established traceability platforms, but they mainly focus on food production enterprises, including meat, vegetables, Chinese herbal medicines, and alcohol (https://zycpzs.mofcom.gov.cn/, accessed on 15 March 2022.). For this reason, China has issued a policy to accelerate the construction of a traceability system for important products, proposing to establish a traceability system at the national level. In 2017, the National Agriculture Food Quality Safety Traceability Management Information Platform (National Traceability Platform, NTP, http://www.qsst.moa.gov.cn/, accessed on 21 August 2021) was activated [24]. In 2018, the NTP was fully promoted in China. Green food, organic agricultural products, and agricultural products with geographical indications identified by the agricultural sector are all included in the traceability management [25]. However, small-scale tea farmers rarely participate in the NTP. It is estimated that 80% of China’s tea production is produced by small-scale tea farmers [2]. Their extensive participation is, therefore, important for tea safety.

The NTP can directly ensure food safety and quality, and reduce the time and cost of food recalls [14]. Participating in the NTP can also indirectly bring environmental, economic, and social benefits. First, it can regulate pesticide and fertilizer inputs for tea farmers [26]. Excessive use of pesticides and fertilizers can lead to soil compaction and water pollution, ecological imbalance in tea gardens, and ultimately limit tea tree growth and tea quality [27,28]. Simultaneously, carbon dioxide from tea cultivation and processing is one of the important sources of agricultural greenhouse gas emissions [29]. By regulating the production behavior of small-scale tea farmers, carbon emissions can be reduced, and the resulting adverse effects of climate change can be mitigated. Furthermore, the traditional cultivation and management of tea has been a major threat to biodiversity, and production based on agricultural standards is beneficial for biodiversity conservation [30]. Eventually, tea farmers produce according to certain standards, which not only can shorten the supply chain by reducing the distance between tea farmers and consumers, but also increase the value chain, thus improving the livelihoods of tea farmers and promoting rural development [31].

Agricultural safety events are typically characterized by immediacy, high uncertainty and complexity [32]. Ensuring the safety of agricultural products must be an action of the whole society, including farmers, companies, consumers and governments [33]. The NTP is considered to be an effective means of guaranteeing the quality and safety of agricultural products [34]. This is because it can identify the responsible parties and thus reduce market failures caused by information asymmetries and unspecified responsibilities. Farmers are both producers and direct participants in the traceability of agricultural products. Studying the behavior of farmers has become the key to solving the problem of agricultural safety. However, the difficulty in promoting the NTP is also for farmers, as they are large and dispersed throughout China [35].

Meanwhile, farmers’ perceptions of new agricultural technologies, including the NTP, are complex. Any policy that requires farmers to adopt new technologies needs to understand their perceptions of the status quo and new technologies [36], especially for small-scale farmers. Because they are already exposed to vulnerability, they may be less willing to take risks [37]. If farmers adopt new technologies, they first need to believe that these technologies will bring them considerable income [38]. When people have a positive attitude toward a new technology, they are mentally prepared for its adoption. This psychological preparation may, in turn, lead them to have a higher willingness to use it [39]. Therefore, by understanding the perceptions of small-scale tea farmers, policymakers can formulate precise policies and accelerate the effective promotion of the NTP in the agricultural sector.

The study was therefore conducted to investigate the perceptions of small-scale tea farmers about the NTP in a typical agricultural area in central China. Based on their different perceptions, we have tried to classify them into different categories of tea farmers. Our research can further expand the scope of research on farmers’ behavior. At the same time, by understanding farmers’ perceptions, the results can be used to support the development of more appropriate policies to promote the NTP in the agricultural sector. Knowledge of farmers’ perceptions will increase our understanding of which policies are more likely to be accepted and therefore adopted by farmers.

## 2. Materials and Methods

### 2.1. Description of National Traceability Platform

**Figure 1:**
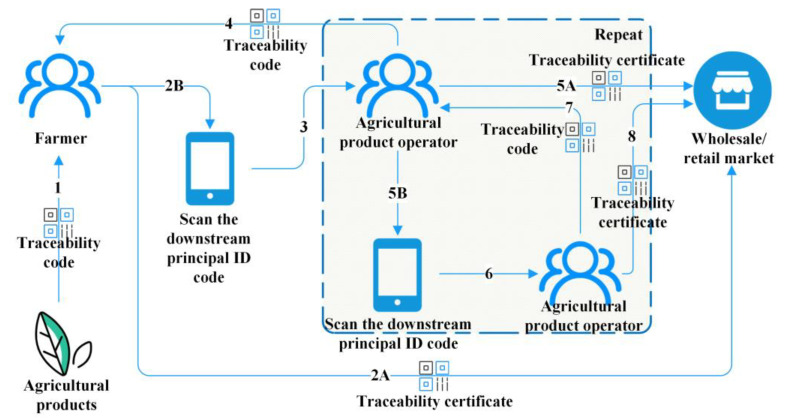
The flowchart of the traceability system.

The national traceability platform was developed and constructed by the Agricultural Product Quality Safety Center of the Ministry of Agriculture and Rural Affairs of PRC, including traceability, supervision, detecting, and enforcement systems. The traceability system is the main component of the NTP, which is used to support agricultural product producers and operators to collect production and circulation information. Figure 1 shows the flowchart of the traceability system. First, farmers put agricultural product information into the system. Second, agricultural products can be sold in circulation and market. Among them, 2A: The agricultural products are directly sold in the market, and the traceability certificate is generated by the system; 2B: Farmers scan the identity codes of downstream transaction subjects to determine their identity information. Third, by scanning the identity code of the downstream transaction subject, farmers fill in relevant transaction information to complete the circulation sales of agricultural products. Fourth, the downstream transaction subjects confirm the completion of the transaction, and the system generates the product traceability code. Fifth, after the downstream subjects confirm the information of the purchased agricultural products and complete the filing, the agricultural products can be selected for circulation sales and market sales. Among them, 5A: Agricultural products are directly sold in the market, and the traceability certificate is generated by the system; 5B: Select the circulation link and repeat the 2B-4 process (This link can be repeated in a loop).

According to the requirements of the Chinese agricultural department, green food, organic agricultural products, and agricultural products with geographical indications (including tea) are all included in the traceability management system. Products not only need to be launched with certificates and codes but also have traceability labels posted on the market [25]. For tea farmers, they need to record variety information, production area environmental information, tea garden reclamation information, soil management information, drug fertilization information, fresh-leaf picking information, testing information, and processing information on the NTP to promote the traceability of the production process [26].

### 2.2. Study Area

**Figure 2:**
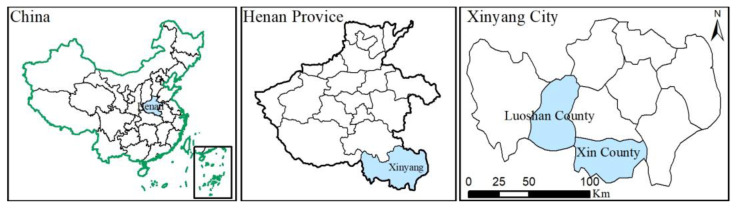
Location of the study area.

Our study was carried out in Xinyang City, Henan Province, Central China (Figure 2), with geographic coordinates ranging from 113°45′ to 115°55′ E and 31°23′ to 32°24′ N. The topography of Xinyang is high in the south and low in the north, and it is located in the transition zone from subtropical to warm temperate. It has a distinct monsoon climate, which is characterized by abundant sunshine and rainfall. In 2020, the total population of Xinyang city was 6.23 million, including 600,000 tea farmers. Its GDP is about $40 billion, of which the tea output value exceeds $1.7 billion [40]. The tea of Xinyang is exported to more than 20 countries and regions around the world [41]. Tea planting covers the entire territory of Xinyang City. The area of tea plantations is equal to 110.37 thousand hectares, with an average annual output of about 70,000 tons of tea. Tea plantation area and output account for 98% and 99% of Henan Province, respectively [42]. There are three main reasons why we chose this area for our research. First, Xinyang is an important green tea production area. Xinyang Maojian Green Tea is one of the top ten famous teas in China, with a brand value of over $1 billion. Secondly, Xinyang has a long history of tea planting, and the tea there is primarily planted by small-scale tea farmers. In addition, Xinyang Maojian Green Tea obtained the National Agricultural Product Geographical Indication Certification in 2020, and tea farmers were included in the NTP for management. However, NTP has not been widely adopted by small-scale tea farmers. Therefore, exploring small-scale tea farmers’ perception of the NTP can not only help us understand the actual needs of tea farmers, but also has significance for the tea industry management that aims to promote the NTP.

### 2.3. Research Methods

#### 2.3.1. Q Methodology

Based on non-positivist philosophy and epistemology, the Q methodology avoids the fallacies of popular positivist research methodology [43,44]; it can scientifically translate subjective human views into objective results [45]. First, unlike other social research methods, it provides numerical results to support the points explained, thus combining the advantages of quantitative and qualitative methods [46,47]. Second, the method is exploratory, meaning it does not focus on the frequency and distribution of research opinions in the population, but rather on diversity [48]. Moreover, it can mitigate certain response biases because respondents are required to engage explicitly with opinions that they might deem inappropriate or unexpected [46]. In recent years, the Q methodology has become an important method for analyzing agricultural issues, including food safety [49], commercial-scale beef farmers [50], intensive agriculture [51], agricultural best management practices [52], wireless sensor network applications [53], and precision farming [54].

**Figure 3:**
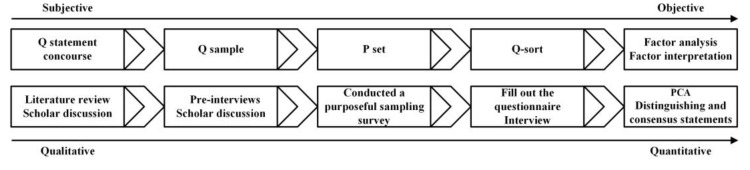
Framework of conducting Q methodology research.

We use the Q methodology to explore small-scale tea farmers’ perceptions of the NTP. The Q methodology mainly consists of four steps (Figure 3): (1) Creating the statement concourse, (2) Selecting the Q sample, (3) Identify respondents (P-set) (the Q samples were sorted according to their viewpoints (Q-sort), and interviews were conducted), (4) Factor analysis was performed on the Q-sort to determine the respondents’ main perceptions. The analysis step used Ken-Q analysis (https://shawnbanasick.github.io/ken-q-analysis/, accessed on 18 January 2022), version 1.0.6.

The first step was creating the statement concourse. A statement concourse can use different sources of information, such as literature, interviews, expert opinions, blogs, and newspapers [47]. By sorting out the relevant literature on the research topic of farmer technology adoption, we summarized the relevant statements in combination with tea planting practices. These statements were examined, discussed, and categorized by a scholar in the field of agricultural economics research and two doctoral students. Finally, 80 statements made up the statement concourse.

The second step involved selecting the Q sample. In general, the Q sample is between 40 and 80 samples large, which are selected from the statement concourse [44]. We conducted pre-interviews with seven tea farmers and removed repeated or ambiguous statements (Table A1). In addition, our team once again discussed these statements. Finally, 48 statements made up the Q sample.

**Figure 4:**
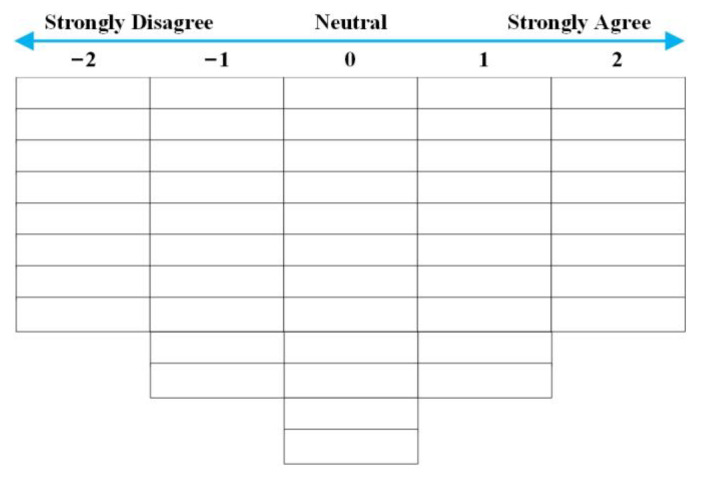
Q-sort grid of standard normal distribution.

The third step was identifying the respondents and conducting the Q-sort. Since Xinyang belongs to a mountainous area, tea farmers live in scattered places. We interviewed 16 respondents face-to-face in two separate sessions. In January 2022, we interviewed ten tea farmers in Balifan Town, Xinxian County, which is known as “the first township of tea in the Central Plains.” In February 2022, we interviewed six tea farmers in Zhutang Town, Luoshan County, which is known as the “Landscape Tea Township”. The two places have a long history of tea, and both are mainly grown by small-scale tea farmers. Our goal was to give tea farmers a clear and pertinent view of the research topic and avoid samples that were too homogeneous. Unlike traditional positivist sampling methods, we conducted a purposeful sampling survey. Tea farmers come from different regions and have different characteristics, such as age, planting experience, education level, and tea planting area. After we explained the research purpose and procedures to them, they were willing to participate in the Q-sort. A Q-sort is an improved rank-sorting technique. Under certain conditions, participants rank question items according to their personal perceived importance [55]. Each tea farmer needed to read the randomly numbered statements and fill in the number of all statements in the grid according to their level of agreement (−2 = “strongly disagree”; −1 = “disagree”; 0 = “neutral”; 1 = “agree”; 2 = “strongly agree”) (Figure 4). After the Q-sort, each tea farmer was interviewed to learn about their thoughts regarding the Q-sort. In such cases, it is relatively free of certain psychological biases such as the dominance effect [46,56].

Fourth, a Q factor analysis and interpretation was conducted. We applied principal component analysis (PCA) to the Q-sort correlation matrix, simplifying the data to several factors. Factors are usually rotated to make the data structure clearer and more interpretable [46]. We used Varimax to rotate the factors. Meanwhile, the Q-sort of these factors was identified and marked in order to reveal differences between factors. Mark [57] refers to the selection of the Q-sort for inclusion in the final factor. In addition, a statement score is calculated for each factor. Based on the statement scores between the different factors, we identified distinguishing and consensus statements. Finally, we highlighted significant distinguishing and consensus statements for each factor. Through the analysis of tea farmer reviews, we, thus, strengthened the quantitative interpretation of these factors.

It is noteworthy that the factor extraction process depends on the eigenvalues and the factor loadings. In other words, not only should the extracted factors have eigenvalues greater than one but also they should include at least two significant viewpoints. There are three types of viewpoints: insignificant, significant and confused. Insignificant viewpoints are those insignificantly correlated with the selected factors. Significant viewpoints are those significantly correlated with one selected factor. Confused viewpoints are those significantly correlated with at least two selected factors. Insignificant and confused viewpoints are excluded from the analysis [44]. Moreover, taking into account each factor, the viewpoints with a loading factor greater than 2.58 × SE (Standard Error) are significant at the 0.01 level. SE is calculated as follows: $SE=1/\sqrt{N}$, where N indicates the number of statements.

An unusual feature of Q methodology is that the rule of thumb that larger sample sizes are better does not necessarily apply [44]. Powerful Q results can be obtained with very small samples [46]. In addition, Milakis et al. [58] and Jang and Lee [59] used the Q methodology to collect 17 and 16 samples for related studies, respectively. Therefore, the samples used in our study can be considered suitable.

#### 2.3.2. Discriminant Analysis

We assessed the robustness of the Q results by using discriminant analysis. Discriminant analysis is a method of modeling dependent variables according to their relationships with certain independent variables [60]. The results are usually presented in the form of a graph, where the categories are projected into the discriminant function [61]. Discriminant analysis is appropriate when the dependent variable is determined in advance [62]. To verify the classification results obtained by the Q factor analysis, we performed Fisher’s linear discriminant analysis using the demographic and economic characteristics of the tea farmers, such as age, education level, gender, and other variables, to determine the group membership of each category of tea farmers.

## 3. Results

### 3.1. Sample Features

The average age of tea farmers was 37.9 years; the oldest and youngest were 60 and 22 years old, respectively (Table 1). The average planting years was 15.9 years; the maximum and minimum planting years were 33 and 2 years, respectively. We included both young and experienced tea farmers. Therefore, we considered our selection of samples to be diverse. They are small-scale tea farmers with an average family tea garden area of 6 mu. They are mostly male. Their average education level is high school; few tea farmers join cooperatives; most tea farmers are engaged in other jobs besides tea planting; their average annual household income is $12.409 dollars.

### 3.2. Q Factor Results

According to the differences in the perception of small-scale tea farmers on the NTP, we divided them into four categories, representing four factors, respectively. These four factors explained 52% of the total variance (Table 2). Among the 16 tea farmers, 14 were significantly loaded factors, and two farmers (13 and 15) did not load any factors. The sample characteristics of each factor are shown in Appendix A Table A2. The four factors show high composite reliability and low standard error.

**Figure 5:**
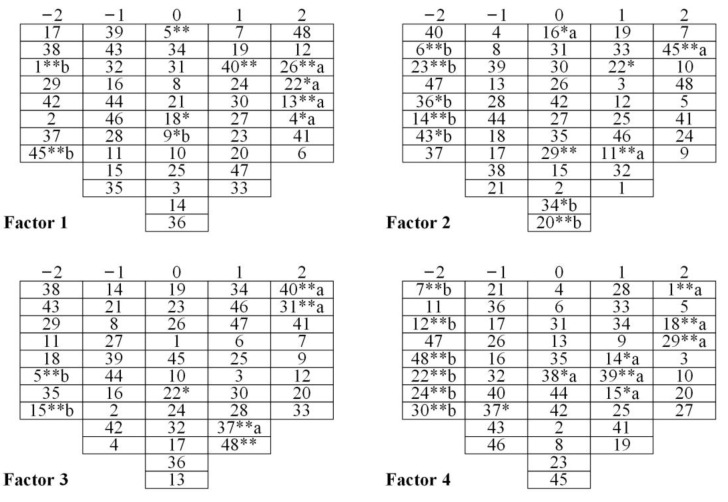
Q-sort of four factors. Note: * Distinguishing statement at *p* < 0.05; ** Distinguishing statement at *p* < 0.01. a: Z-Score for the statement is higher than in all other factors; b: Z-Score for the statement is lower than in all other factors.

Figure 5 shows the Q-sort of the four factors. Tea farmers within each factor had similar perceptions of the NTP. We use factor 1 as an example to explain how to read the Q-sort. Among them, statements 48, 12, 26, 22, 13, 4, 41, and 6 in factor 1 represent “strongly agree.” Statements 26 and 13 are significantly different from the other factors at the 1% level, and statements 22 and 4 are significant at the 5% level. Appendix A Table A3 lists the full Q-sort for each factor.

Distinguishing and consensus statements can be used to reveal differences and similarities between factors [63]. To better understand the perceptions of small-scale tea farmers, we combined their comments made in the interviews to describe the distinguishing statements (Table A3) (Section 3.2.1, Section 3.2.2, Section 3.2.3 and Section 3.2.4) and consensus statements (Section 3.2.5). For each factor, the numbers in parentheses represent the ordinal number of the statement and its Q-sort value. For factor 1, (26, 2) is represented in the position of 2, meaning the response to statement 26 was considered to be “strongly agree.”

#### 3.2.1. Factor 1: Active Participation

**Figure 6:**
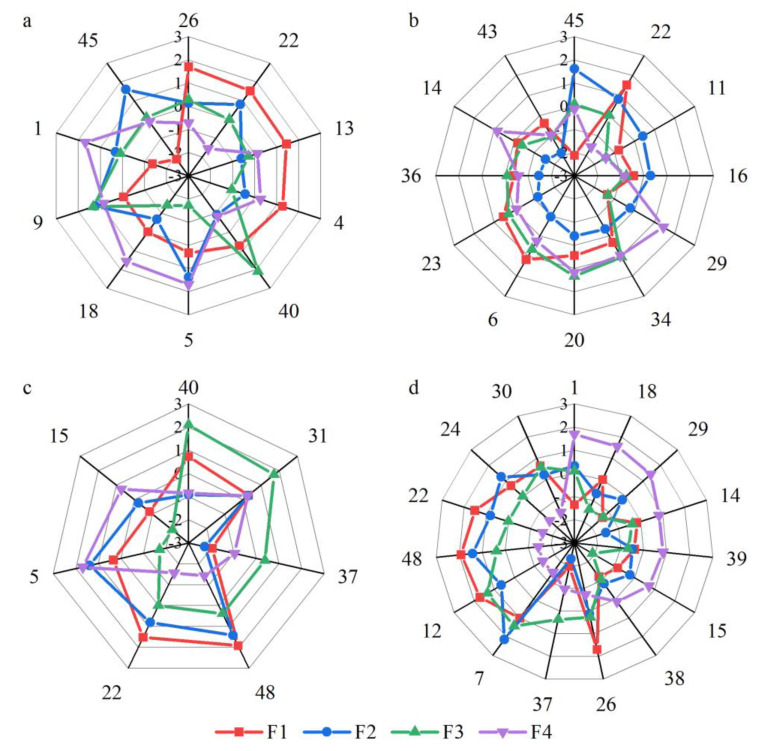
Average loadings for the distinguishing Statements of four factor. Note: The numbers in the Figure represent the order of the statements.

Results showed that factor 1 accounted for 21% of the overall variance. Five tea farmers’ viewpoints fell into this factor (Table 2). These tea farmers were very willing to spend time and energy on the NTP (45, −2) as they believed that the cost of participating in the NTP was not high (1, −2) (Figure 6a). “I like to try new things, including participating in the NTP, even if I pay some costs,” said Tea Farmer 7. Furthermore, if participating in the NTP was in line with their principles, values, and beliefs (4, 2), they would continue to focus on the development trend of the platform (26, 2). As Tea Farmer 5 said: “The government’s requirements for tea production are becoming more and more stringent; participation in tea traceability is a trend.” Meanwhile, they supported participation in the NTP to reduce the adverse environmental impacts of tea cultivation (40, 1). However, they maintained the neutral attitude that participating in the NTP could reduce the risk of tea cultivation (5, 0) and sell tea to the national market (9, 0). Tea Farmer 11 explained: “If tea cultivation is carried out in accordance with national standards, we will reduce the use of pesticides and fertilizers, which will be of benefit for the environment. But tea growth will face pests and diseases, which may increase the risk of planting.” In addition, tea farmers believed that they needed to spend a long time learning the relevant knowledge and technology related to the NTP (22, 2). “I need to spend time learning how to use it because I don’t understand the NTP,” said Tea Farmer 10. In general, such tea farmers were willing to bear the cost, and they had a strong willingness to participate in the NTP. Therefore, such tea farmers demonstrated the characteristics of active participation.

#### 3.2.2. Factor 2: Resistant Participation

Five of the tea farmers fall into factor 2, which explains 12% of the total variance (Table 2). Such tea farmers strongly agreed that participating in the NTP cannot increase tea sale revenue (6, −2) (Figure 6b). They didn’t want to spend a lot of time and energy learning how to use technology and participating in the NTP, so they might as well go out to work and make money (11, 1) (22, 1) (45, 2). Tea Farmer 4 said: “I need to go out to work to earn more money to improve my family’s living standard. It is difficult to maintain my family’s livelihood by relying on tea income alone.” They strongly disagreed with the influence of subjective norms (43, −2) (23, −2). Tea Farmer 3 explained: “I have been growing tea for 30 years, and it is hard for other people’s opinions to influence me.” They also lacked the necessary equipment to participate in the NTP, such as an “office computer + scanner” or “smartphone + printer” (36, −2). Tea Farmer 8 said: “I only have a mobile phone, and the network signal is poor. I don’t have any other devices.” To sum up, such tea farmers strongly believed that the costs and risks of participating in the NTP far outweighed the benefits, and it was rather difficult for subjective norms to influence their perceptions. Consequently, such tea farmers showed the characteristics of resistant participation.

#### 3.2.3. Factor 3: Risk Aversion

Factor 3 contains two tea farmers and they represented 10% of the overall variance (Table 2). Such tea farmers strongly supported participation in the NTP to reduce the adverse environmental impacts of tea cultivation (40, 2), and they were concerned about the risks of tea cultivation (5, −2) (Figure 6c). Tea Farmer 14 said: “I pay attention to environmental issues because I can also benefit from it. But I am more worried about the risks of tea cultivation, which directly affects my income.” Meanwhile, they believed that tea safety was the most basic step in food safety production (31, 2). In addition, tea farmers believed that the government supported their participation in the NTP (48, 1). They also had the funds to purchase equipment to participate in the NTP (37, 1). Tea farmer 16 said: “In recent years, I can feel that the government has formulated many policies for the development of tea.” However, they would not feel guilty if they did not participate in the NTP (15, −2). In brief, they could feel the benefits of participating in the NTP, including environmental and tea safety. Risk perception had a greater impact on their participation in the NTP. For this reason, these tea farmers showed the characteristic of risk aversion.

#### 3.2.4. Factor 4: Being Pressure-Driven

Factor 4 explains 9% of the total variance and two tea farmers’ viewpoints fell into this category (Table 2). Such tea farmers strongly believed that the cost of participating in the NTP was too high (1, 2). They felt that the government did not support their participation in the NTP (48, −2) (Figure 6d). They also felt no obligation (30, −2) and no confidence to participate in the NTP (24, −2). Tea Farmer 6 explained: “If I participate in the NTP, I need to pay for the related equipment. I feel that the government should bear these costs.” Meanwhile, if neighbors were willing to participate in the NTP, they were also willing to try it (18, 2). They strongly agreed that participation in the NTP was a matter of social pressure (29, 2). Tea Farmer 12 said: “If all the neighbors participated, I would also participate.” In addition, they believed that participating in the NTP cannot improve the quality and safety of tea (7, −2). They also disagreed with raising awareness of tea farmers’ participation in the NTP in order to ensure tea safety (12, −2). In summary, such tea farmers were greatly affected by social pressure, and policies also affected their behavioral choices. Thus, these tea farmers have the characteristic of being pressure-driven.

#### 3.2.5. Consensus Statements

Consensus statements are those that do not distinguish between factor differences. Seven of the 48 statements were consensus statements (Table 3), and all were significant at the 5% level. Tea farmers supported the relevant measures of the NTP, and they also believed that participating in the NTP would make tea sales more convenient. If the conditions were right, they would consider participating in the NTP. Moreover, tea farmers believed that participating in the NTP did not necessarily improve the efficiency of tea planting, but that it might increase the cost of planting. The probable reason was that the NTP was new to most tea farmers, and they all felt that participation might bring certain risks. In addition, tea farmers did not believe that agricultural technicians supported their participation in the NTP. Tea Farmer 11 explained: “Nowadays, we rarely see agricultural technicians; it is difficult to get help from them.”

### 3.3. Robustness Check

Table 4 lists the Fisher discriminant analysis results. The discriminability of Function 1 is statistically significant, which confirms that there is a significant difference between categories. The eigenvalue of Function 1 is 79.43, the variance is 93%, and the correlation coefficient is positive and relatively large (0.99). The statistical significance of each discriminant function was evaluated according to Wilks’ Lambda. The range of this parameter is 1.0 (no discrimination) to 0 (perfect discrimination). The Wilks’ Lambda values of Function 1 and Function 2 are 0 and 0.07, respectively, which means that there are significant differences between different categories of tea farmers.

**Figure 7:**
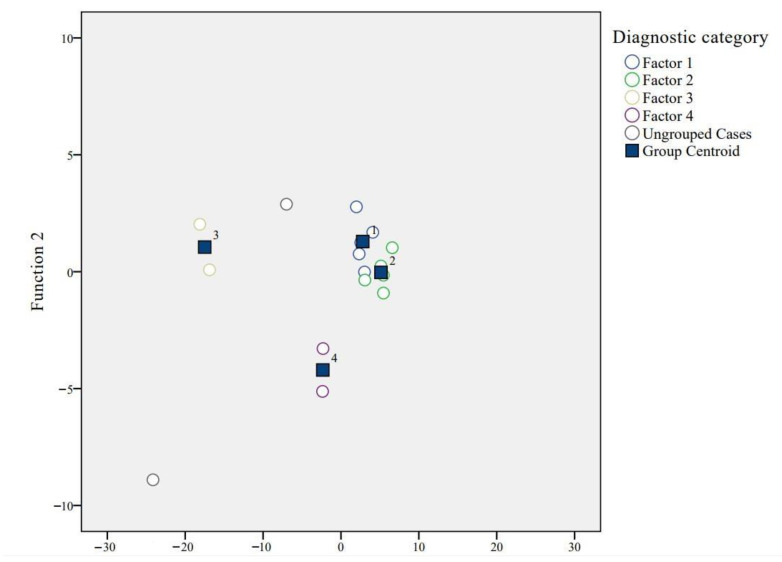
Groupings of tea farmers using Functions 1 and 2.

Figure 7 plots the grouping of Functions 1 and 2, using their discriminant coefficients as coordinates for all tea farmers. It is easy to see that the classification results show smaller within-group distances and larger inter-group distances. Therefore, it can be stated that the results obtained by the Q-factor analysis are robust. That is, the Q-factor analysis can effectively predict and classify the perception of small-scale tea farmers for the NTP.

Table 5 shows the classification results of discriminant analysis. Through the prediction of the discriminant function, we find that all observations can be effectively predicted. That is, the classification results of tea farmers obtained by the Q analysis are the same as those obtained by discriminant analysis. Therefore, 100% of the original group of tea farmers are correctly classified.

It is noteworthy that this study developed the initial Q sample based on an extensive literature review, and subsequently invited four experts and doctors to provide comments and suggestions to improve the initial Q sample. Therefore, this study adequately addressed the content validity requirements. Moreover, each step of this study, from the identification of Q samples and selection of P sets to Q sorting and factor analysis, strictly follows the standard procedure of Q methodology, and thus the reliability of this study is ensured. Finally, if a future study investigating a larger population revealed other perspectives, the results would strengthen, rather than weaken, the results of this study, as the future study further demonstrates the large variance of opinions in the population.

## 4. Discussion

The Q methodology can be a useful method for answering the question “What are farmers’ real perceptions of the NTP”? Not only did we reveal the perception of difference and similarity among small-scale tea farmers, but we also overcame the limitations of qualitative and quantitative methods in studying farmers. In the context of agricultural safety, we believe that the Q methodology can add color to research in the area of farmer perception.

By analyzing the Q-factor results, we identified four different categories of tea farmers, representing their different perceptions of the NTP. Four categories of tea farmers explained 52% of the total variance, and the related findings of Kroesen and Bröer [64] and Krabbenborg et al. [65] had an explanatory power of 49% and 40%, respectively. Therefore, we considered the findings to be credible. Research showed that there are perceptual differences among small-scale tea farmers, which was consistent with Oyinbo et al. [66], who also acknowledge significant heterogeneity among farmers. Meanwhile, on some specific issues, tea farmers have similar perceptions.

The first category of tea farmers were those characterized by active participation. They are willing to bear the cost and put their time and energy into the NTP. Farmers believed that there was a cost to adopting new agricultural technologies. They would, therefore, invest in it only if the expected benefits outweighed the costs [67]. In fact, tea production faced many challenges, including price fluctuation, climate change, and cultivation risks [22,68]. For small-scale tea farmers, participating in the NTP was a strategic choice, which might reduce planting costs and grant access to a broader market. They believed that it took a long time to learn relevant knowledge and technology simultaneously. The survey found that local tea farmers rarely understood the NTP; thus, they needed to learn how to use it.

The second category of tea farmers were those who engaged in resistant participation. They did not want to spend time and energy on the NTP but to go to work and make money instead. This result is also confirmed by Li et al. [69], who showed that tea farmers face cost risk, time risk, and opportunity risk. With the rapid development of the social economy, a large part of the income of rural households comes from other employment rather than agricultural production [70]. This is also one of the main reasons why tea farmers were reluctant to participate in the NTP. Meanwhile, it was difficult for subjective norms to affect the perception of tea farmers. Such perceptions being external and self-perception being internal, the impact of self-perception was more direct. Farmers relied on their previous experience in managing production [67]; thus, it was difficult for subjective norms to influence their long-term beliefs.

The third category of tea farmers were those who were risk-averse. They could see the benefits of participating in the NTP, including environmental improvements and tea safety. Li et al. [69] argued that perceived benefits can more strongly influence farmers’ adoption of agricultural technologies through positive recognition of the perceived value. This might increase their willingness to participate in the NTP and focus on consumer health. But tea farmers were more worried about planting risks. Most farmers were risk-averse [71]. Because of the conflict between economic goals and environmental goals, they felt no guilt for not participating in the NTP. This showed that tea farmers’ perception of the NTP was only superficial as it had not been fully understood and accepted.

The fourth category of tea farmers were those who were pressure-driven. They were strongly influenced by social pressure. Kallgren et al. [72] argued that individuals followed social norms because they not only feared social pressure but also used it as a source of information to understand which behavior is appropriate in a particular situation. Thus, social norms played a key role in farmer technology adoption [73]. The more positively tea farmers evaluated the NTP, the higher their willingness to participate would be. Furthermore, they did not have the confidence to participate in the NTP. Ajzen [74] found that people’s behavior was strongly influenced by their self-confidence. A possible reason for this was that farmers lacked confidence in understanding and using new technologies due to the lack of effective policy support [75].

Furthermore, tea farmers had similar views on some specific issues. For example, they were all concerned that participating in the NTP might increase the cost of tea cultivation. Lou et al. [76] believed that tea farmers were affected by realistic uncertainties and past habits when adopting agricultural technologies. They were all small-scale growers and had no experience participating in the NTP. If they participated in the NTP, they needed to consider its potential risks, future benefits, and opportunity costs from traditional to standard tea cultivation. Unlike food crop production, tea cultivation was rarely subsidized by the government, which made it less resilient to risks. Tea farmers also felt that they were not supported by agricultural technicians. Diaz et al. [77] argued that the opinions, views, and comments of agricultural technicians were important to farmers, which would influence their adoption and decision-making. As far as we know, local technology promotion efforts were low, and it was difficult for tea farmers to directly refer to the opinions of agricultural technicians.

The recently amended Law of the People’s Republic of China on Quality and Safety of Agricultural Products will implement traceability management for agricultural products listed in the Agricultural Products Quality and Safety Traceability Catalogue. Meanwhile, the protection and management of geographical indication agricultural products will be strengthened. This shows that traceability of agricultural products is indeed the urgent issue at present.

## 5. Conclusions

This study provides a new contribution to the promotion of the NTP. We have focused all our attention on the perceptions of small-scale farmers, and the findings could provide new evidence for the debate on the NTP. Meanwhile, new perspectives and insights on the subject are provided for policy makers to rethink the promotion policy of the NTP. In addition, we encourage action to prevent the negative impact of the promotion of the NTP on farmers.

There is an increasing amount of research on the NTP in various areas, but not much effort seems to have been made on the farmer side. The NTP are an important means of ensuring the safety of agricultural products. Farmers are both producers and direct participants in the NTP. Farmers’ perception of the NTP is a prerequisite for their adoption. Due to a lack of understanding of farmers’ perceptions, policy makers may have adopted the wrong extension policies.

This study of small-scale Chinese tea farmers’ perceptions of the NTP further expands the scope of research on farmer behavior. The study shows that there are four categories of tea farmers with different characteristics, including active participation, resistant participation, risk aversion, and driven by pressure, and each category contains tea farmers with very similar perceptions. The first category can actively bear the cost; thus, they should be the first to participate in the NTP. The traditional perception of the second category is difficult to change in the short term, so they may be the last category of participants. The third category may participate in the NTP if planting risks can be reduced. Under social pressure, the fourth category may also participate in the NTP. Although there is significant heterogeneity in the perceptions of tea farmers, some commonalities are also highlighted. They all worry that participating in the NTP may increase planting costs and risks, and find it rather difficult to get support from agricultural technicians. Therefore, understanding the different perceptions of tea farmers is a prerequisite for formulating precise policies. Our findings can serve as the basis for the promotion of the NTP in the agricultural sector.

### Recommendations and Study Limitations

Planning should be based on an overall perspective and implement tailored policies. For the first category of tea farmers, we suggest carrying out platform operation training from the perspective of meeting the needs of tea farmers, formulating training programs scientifically, and improving the pertinence and timeliness of the training offered. Self-service learning platforms and technical support should also be provided to improve the ability of tea farmers to master the business processes, system functions, and related operations of the NTP. For the second category, the government widely publicize the application progress and effectiveness of the NTP through various forms, including online and offline formats. It is also necessary to popularize traceability knowledge and cultivate traceability culture more deeply to positively influence the cognitive structure of tea farmers. For the third category, we recommend holding regional expositions and promotion meetings to publicize and display traceable products so as to increase the awareness of tea farmers about traceable products. The government can also actively promote the entry of tea into large supermarkets and e-commerce and promote the connection between production and sales of high-quality tea in order to increase the enthusiasm of tea farmers to participate in the NTP. For the fourth category, we recommend strengthening policy support, especially in terms of increasing funds to subsidize the necessary equipment expenditures for tea farmers to participate in the NTP. Policies should also support the establishment of standard tea gardens that demonstrate the use of the NTP to increase tea farmers’ awareness of the NTP. In addition, the government should exert the appeal and influence of large-scale tea farmers to enhance their confidence in participation as well. Finally, for all tea farmers, we suggest using a variety of ways to help them understand and recognize the benefits of the NTP, including the agricultural sector, NGOs, and agricultural colleges. The government can also consider establishing a risk compensation and price guarantee mechanism for tea traceability to reduce planting risks and promote steady growth in the income of tea farmers.

Although we conducted a purposeful survey of small-scale tea farmers, it was still difficult to ensure that all tea farmers’ perceptions of the NTP were explored. The study may have missed some points, such as two tea farmers who did not belong to any of the four categories outlined in this study. We should also acknowledge some limitations. First, there were few participants. Future studies could increase the sample size to include farmers at different times and levels of participation. Moreover, since this study focuses on perceptual identification, follow-up studies could also conduct quantitative surveys to assess the perceptual distribution and driving factors. Furthermore, this study takes tea farmers in Xinyang City as an example, and the applicability of the findings to farmers of different crops and in different regions is unclear. Future research can, therefore, expand the scope of study to different types of farmers and add more samples with different regional attributes. Eventually, future research should also be extended to other stakeholders to explore their perceptions.

## Figures and Tables

**Table 1:** Descriptive Statistics.

Variable	Description	Mean	SD	Min	Max
Gender	1 = Male, 0 = Female	0.69	0.479	0	1
Age	Actual age (years)	37.94	10.963	22	60
Marital status	1 = Unmarried, 2 = Married, 3 = Divorced, 4 = Widowed	1.75	0.447	1	2
Education level	1 = Primary school, 2 = Middle school, 3 = High school, 4 = College and above	3	0.894	2	4
Planting years	Tea planting years	15.88	9.135	2	33
Household size	Number of household members	4.75	1.183	3	7
Tea garden area	Tea garden planting area (mu)	6.06	4.419	2	20
Number of employees		0.81	1.109	0	3
Whether to join a cooperative	1 = Yes, 0 = No	0.13	0.342	0	1
Are you engaged in other employment	1 = Yes, 0 = No	0.87	0.342	0	1
Annual household income	Thousand US dollars	12.409	6.575	1.513	45.4

**Table 2:** Factor matrix for all Q-sorts (four factors after rotation).

Q-Sort	F1	F2	F3	F4
1	−0.20	0.53 *	−0.16	0.00
2	0.18	0.44 *	0.36	0.14
3	0.12	0.77 *	−0.04	−0.14
4	0.33	0.42 *	0.07	−0.08
5	0.60 *	−0.18	−0.04	−0.49
6	0.18	−0.19	−0.14	0.41 *
7	0.74 *	0.02	−0.09	0.22
8	0.17	0.53 *	0.31	0.19
9	0.55 *	0.12	0.13	−0.11
10	0.74 *	0.01	−0.06	0.00
11	0.67 *	0.42	0.10	0.12
12	−0.26	0.16	−0.07	0.84 *
13	0.49	0.17	0.50	−0.12
14	−0.04	0.15	0.65 *	−0.01
15	0.33	−0.13	0.44	0.44
16	−0.15	−0.23	0.78 *	−0.22
Eigenvalues	3.31	1.85	1.66	1.36
% Explained Variance	21	12	10	9
Cumulative % Explained Variance	21	33	43	52
Number of defining sort	5	5	2	2
Avg. Rel. Coef.	0.8	0.8	0.8	0.8
Composite Reliability	0.952	0.952	0.889	0.889
S.E. of Factor Z-scores	0.219	0.219	0.333	0.333

Note: * *p* < 0.05.

**Table 3:** Consensus statements across all factors (Q-Sort Value).

No.	Statement	F1 Q-SV	F2 Q-SV	F3 Q-SV	F4 Q-SV
8	Participating in the NTP can improve the efficiency of tea cultivation	0	−1	−1	0
17	Even if the cost of tea cultivation increases, I will participate in the NTP	−2	−1	0	−1
19	If the conditions are right, I intend to participate in the NTP	1	1	0	1
25	I will participate in the NTP	0	1	1	1
33	Participating in the NTP will make tea sales more convenient	1	1	2	1
41	I support the relevant measures of the NTP	2	2	2	1
44	Agricultural technicians supported me to participate in the NTP	−1	−1	−1	0

**Table 4:** Fisher discriminant analysis results.

Function	Eigenvalue	% of Variance	Cumulative %	Canonical Correlation	Wilks’ Lambda	Chi-Square	df	Sig.
1	79.43	93.0	93.0	0.99	0.00	45.40	27	0.01
2	4.59	5.4	98.4	0.90	0.07	16.88	16	0.39
3	1.40	1.6	100.0	0.76	0.41	5.70	7	0.58

**Table 5:** Classification results of the Fisher discriminant functions.

Diagnostic Category			Predicted Group Membership				Total
			F1	F2	F3	F4	
Original	Count	F1	5	0	0	0	5
		F2	0	5	0	0	5
		F3	0	0	2	0	2
		F4	0	0	0	2	2
		Ungrouped observations	0	0	1	1	2
	%	F1	100.0	0.0	0.0	0.0	100.0
		F2	0.0	100.0	0.0	0.0	100.0
		F3	0.0	0.0	100.0	0.0	100.0
		F4	0.0	0.0	0.0	100.0	100.0
		Ungrouped observations	0.0	0.0	50.0	50.0	100.0

## Data Availability

The data presented in this study are available on request from the corresponding author.

## Appendix A

**Table A1:** Demographic characteristics of pre-interviewed tea farmers.

No.	Gender	Age	Marital Status	Education Level	Planting Years	Household Size	Tea Garden Area (mu)	Number of Employees	Whether to Join a Cooperative	Are you Engaged in other Employment	Annual Household Income (Thousand US Dollars)
1	Male	30	Married	College and above	10	7	6	0	No	Yes	7.142
2	Female	30	Married	Middle school	2	3	2	2	No	Yes	4.285
3	Female	34	Married	College and above	20	5	5	0	No	Yes	1.428
4	Male	34	Married	Middle school	6	6	2	3	Yes	No	4.285
5	Female	37	Married	College and above	20	7	5	0	No	Yes	14.285
6	Male	49	Married	Middle school	20	3	10	0	No	Yes	17.142
7	Male	50	Married	High school	20	4	3	1	No	Yes	7.142

**Table A2:** The basic characteristics of tea farmers by factors.

Characteristic	Description	F1	F2	F3	F4
Gender	Male	2	4	1	2
	Female	3	1	1	0
Mean of personal characteristic	Age (years)	37.8	41.8	33	39
	Planting years	18	18.4	8.5	21.5
	Household size	4.6	5	4	5
	Number of employees	0	0.8	2.5	0
	Annual household income (Thousand US dollars)	12.4	17.6	5.3	12.1
	Tea garden area (mu)	6.6	7.6	3	7.5
Marital status	Unmarried	1	0	1	1
	Married	4	5	1	1
Education level	Primary school	0	0	0	0
	Middle school	1	1	2	1
	High school	1	2	0	0
	College and above	3	2	0	1
Join a cooperative		0	0	0	1
Engaged in other employment		5	5	2	1

**Table A3:** Q-sort value and Z-score for four factors.

No.	Statement	F1 SV	F1_Z-Score	F2 SV	F2 Z-Score	F3 SV	F3 Z-Score	F4 SV	F4 Z-Score
1	The cost of participating in the NTP is too high	−2	−1.35 **	1	0.32	0	0.10	2	1.69 **
2	The benefits of participating in the NTP are not enough to offset the costs	−2	−1.58	0	−0.33	−1	−0.95	0	−0.13
3	Participating in the NTP is valuable to me	0	−0.15	1	0.71	1	0.57	2	1.18
4	Participating in the NTP is consistent with my principles, values and beliefs	2	1.26 *	−1	−0.44	−1	−1.05	0	0.26
5	Participating in the NTP can reduce the risk of tea cultivation	0	0.33 **	2	1.38	−2	−1.72 **	2	1.69
6	Participating in the NTP can increase the sales revenue of tea	2	1.17	−2	−0.96 **	1	0.67	0	0.26
7	Participating in the NTP can improve the quality and safety of tea	1	1.01	2	2.15	2	1.43	−2	−1.43 **
8	Participating in the NTP can improve the efficiency of tea cultivation	0	0.26	−1	−0.48	−1	−0.57	0	−0.13
9	Participating in the NTP enables tea to be sold to the national market	0	−0.05 *	2	1.25	2	1.33	1	0.84
10	Participating in the NTP is an inevitable trend of future development	0	−0.09	2	1.59	0	0.00	2	1.18
11	Participating in the NTP requires a lot of time and effort to focus on relevant information	−1	−0.79	1	0.41 **	−2	−1.43	−2	−1.43
12	Tea safety is a key issue and I need to raise awareness to participate in the NTP	2	1.70	1	0.64	2	1.33	−2	−1.43 **
13	I will consider the opinions of my neighbors when participating in the NTP	2	1.44 **	−1	−0.61	0	−0.29	0	0.13
14	Participating in the NTP, I feel like being a better person	0	−0.17	−2	−1.57 **	−1	−0.38	1	0.84 *
15	I would feel guilty if not participating in the NTP	−1	−0.84	0	−0.24	−2	−2.10 **	1	0.72 *
16	The functionality of the NTP is clear and I can easily remember how to operate it	−1	−0.44	0	0.28 *	−1	−0.76	−1	−0.84
17	Even if the cost of tea cultivation increases, I will participate in the NTP	−2	−1.06	−1	−0.80	0	−0.10	−1	−0.72
18	If neighbors participate in the NTP, I am also willing to try it	0	−0.01 *	−1	−0.67	−2	−1.43	2	1.56 **
19	If the conditions are right, I intend to participate in the NTP	1	0.93	1	1.15	0	0.29	1	0.46
20	Social media publicity has had an important impact on my participation in the NTP	1	0.44	0	−0.40 **	2	1.33	2	1.18
21	With simple training, I can easily participate in the NTP	0	0.06	−1	−0.85	−1	−0.38	−1	−0.59
22	I will spend a long time learning the relevant knowledge and technology of the NTP	2	1.52 *	1	0.81 *	0	0.00 *	−2	−1.56 **
23	My family encouraged me to participate in the NTP	1	0.54	−2	−1.18 **	0	0.29	0	−0.13
24	I have enough confidence to participate in the NTP	1	0.69	2	1.25	0	0.00	−2	−1.56 **
25	I will participate in the NTP	0	−0.11	1	0.56	1	0.67	1	0.72
26	I will continue to pay attention to the development trend of the NTP	2	1.70 **	0	0.13	0	0.29	−1	−0.72 *
27	I will be influenced by agricultural technicians and participate in the NTP	1	0.56	0	0.00	−1	−0.57	2	1.18
28	I think it will be easy to participate in the NTP for tea sales	−1	−0.75	−1	−0.62	1	0.38	1	0.97
29	I think participation in the NTP is a social pressure	−2	−1.37	0	−0.22 **	−2	−1.33	2	1.43 **
30	I am obliged to participate in the NTP	1	0.63	0	0.22	1	0.57	−2	−1.56 **
31	Paying attention to tea safety issues and participating in the NTP are the most basic steps in food safety production	0	0.30	0	0.27	2	1.72 **	0	0.26
32	Participating in the NTP requires more capital investment	−1	−0.39	1	0.37	0	0.00	−1	−0.84
33	Participating in the NTP will make tea sales more convenient	1	0.39	1	1.14	2	1.05	1	0.97
34	Participating in the NTP for tea sales can save time	0	0.32	0	−0.34 *	1	1.05	1	0.97
35	I would like to participate in NTP in the future	−1	−0.98	0	−0.12	−2	−1.72	0	0.13
36	I have the essential resources to participate in the NTP (“office computer + scanner” or “smartphone + printer”)	0	−0.39	−2	−1.47 *	0	−0.10	−1	−0.59
37	I have funds to purchase equipment for the NTP	−2	−1.96	−2	−2.29	1	0.38 **	−1	−0.97 *
38	I have the ability to grow, produce and sell tea in accordance with the requirements of the NTP	−2	−1.20	−1	−0.82	−2	−1.05	0	0.13 *
39	I should encourage other farmers to participate in the NTP	−1	−0.39	−1	−0.49	−1	−0.67	1	0.84 **
40	I support participation in the NTP to reduce the adverse environmental impact of tea cultivation	1	0.73 **	−2	−0.92	2	2.10 **	−1	−0.84
41	I support the relevant measures of the NTP	2	1.20	2	1.38	2	1.72	1	0.72
42	Tea is a proper agricultural product to participate in NTP	−2	−1.50	0	0.04	−1	−0.95	0	0.00
43	Consumers think I should participate in the NTP	−1	−0.39	−2	−1.87 *	−2	−1.05	−1	−0.97
44	Agricultural technicians supported me to participate in the NTP	−1	−0.69	−1	−0.63	−1	−0.67	0	0.13
45	I would rather go out to work to make money than spend time and energy on the NTP	−2	−2.12 **	2	1.61 **	0	0.10	0	−0.13
46	I would recommend it to others when participating in the NTP	−1	−0.71	1	0.49	1	0.76	−1	−0.97
47	The government has supportive policies for participating in the NTP	1	0.39	−2	−1.28	1	0.76	−2	−1.43
48	The government supports me to participate in the NTP	2	1.92	2	1.42	1	0.38 **	−2	−1.43 **

Note: * Distinguishing statement at *p* < 0.05; ** Distinguishing statement at *p* < 0.01.
